# Supplementary material for: Optical characteristics after Descemet membrane endothelial keratoplasty: 1-year results
Source: PLoS One. 2020 Oct 14;15(10):e0240458. doi: 10.1371/journal.pone.0240458 (PMC7556451; doi:10.1371/journal.pone.0240458)
Supplement: S1 File — (PDF) [file pone.0240458.s001.pdf]

Supplementary Figures

Article title

One-year course of corneal topographic metrics after Descemet membrane endothelial keratoplasty

Journal name

PLOS ONE

Authors' names

Takahiko Hayashi[1,2,3,4,5], Akira Kobayashi[6],

Hidenori Takahashi[3], Itaru Oyakawa[7], Naoko Kato[1,8], Takefumi Yamaguchi[9]

Affiliations

[1] Department of Ophthalmology, Yokohama Minami Kyosai Hospital, Yokohama, Kanagawa, Japan

[2] Department of Ophthalmology, University of Cologne, Cologne, Germany

[3] Department of Ophthalmology, Jichi Medical University, Shimotsuke, Tochigi, Japan

[4] Department of Ophthalmology, Yokohama City University School of Medicine, Kanagawa, Japan

[5] Department of Technology and Design Thinking for Medicine (DT2M), Hiroshima University

[6] Department of Ophthalmology, Graduate School of Medical Science, Kanazawa University, Kanazawa, Japan

[7] Department of Ophthalmology, Heart Life Hospital, Nakagami-gun, Okinawa, Japan

[8] Minamiaoyama Eye Clinic, Minato-ku, Tokyo, Japan

[9] Department of Ophthalmology, Tokyo Dental College, Ichikawa General Hospital, Chiba, Japan

E-mail address of the corresponding author

E-mail: takamed@gmail.com

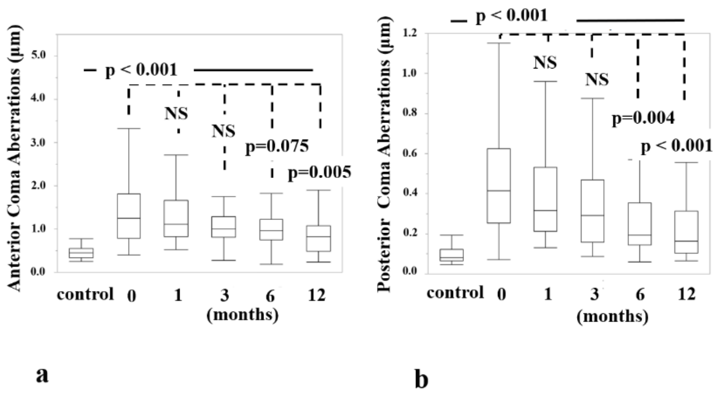
Anterior Coma Aberrations (μm)
p < 0.001
NS
NS
p=0.075
p=0.005
control
0
1
3
6
12
(months)
a
Posterior Coma Aberrations (μm)
p < 0.001
NS
NS
p=0.004
p < 0.001
control
0
1
3
6
12
(months)
b
Total Coma Aberrations (μm)
p < 0.001
NS
NS
p=0.033
p=0.002
control
0
1
3
6
12
(months)
c

S1 Fig.

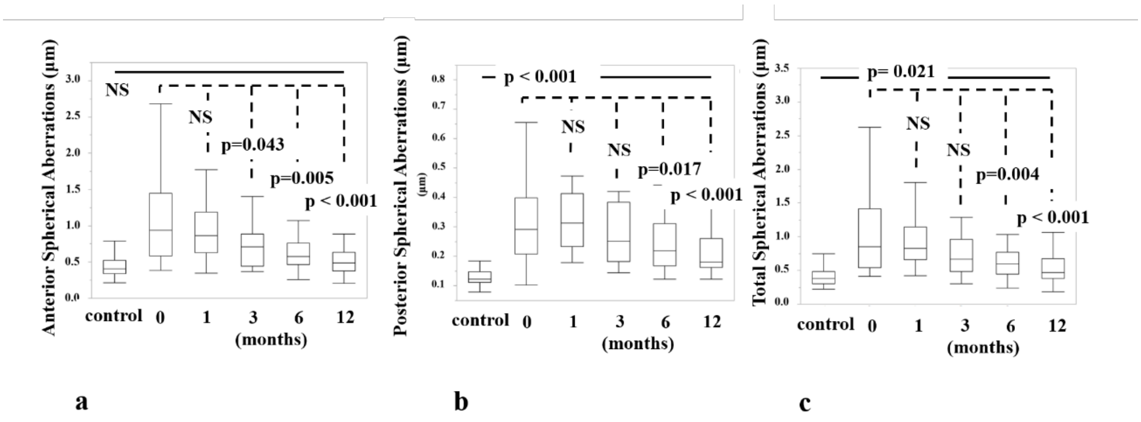
Anterior Spherical Aberrations (μm)
NS
NS
p=0.043
p=0.005
p < 0.001
control
0
1
3
6
12
(months)
a
Posterior Spherical Aberrations (μm)
p < 0.001
NS
NS
p=0.017
p < 0.001
control
0
1
3
6
12
(months)
b
Total Spherical Aberrations (μm)
p= 0.021
NS
NS
p=0.004
p < 0.001
control
0
1
3
6
12
(months)
c

S2 Fig.
