# Supplementary material for: Optical characteristics after Descemet membrane endothelial keratoplasty: 1-year results
Source: PLoS One. 2020 Oct 14;15(10):e0240458. doi: 10.1371/journal.pone.0240458 (PMC7556451; doi:10.1371/journal.pone.0240458)
Supplement: S1 Table — (PDF) [file pone.0240458.s002.pdf]

Supplementary Table

Article title

One-year course of corneal topographic metrics after Descemet membrane endothelial keratoplasty

Journal name

PLOS ONE

Authors' names

Takahiko Hayashi[1,2,3,4,5], Akira Kobayashi[6],

Hidenori Takahashi[3], Itaru Oyakawa[7], Naoko Kato[1,8], Takefumi Yamaguchi[9]

Affiliations

[1] Department of Ophthalmology, Yokohama Minami Kyosai Hospital, Yokohama, Kanagawa, Japan

[2] Department of Ophthalmology, University of Cologne, Cologne, Germany

[3] Department of Ophthalmology, Jichi Medical University, Shimotsuke, Tochigi, Japan

[4] Department of Ophthalmology, Yokohama City University School of Medicine, Kanagawa, Japan

[5] Department of Technology and Design Thinking for Medicine (DT2M), Hiroshima University

[6] Department of Ophthalmology, Graduate School of Medical Science, Kanazawa University, Kanazawa, Japan

[7] Department of Ophthalmology, Heart Life Hospital, Nakagami-gun, Okinawa, Japan

[8] Minamiaoyama Eye Clinic, Minato-ku, Tokyo, Japan

[9] Department of Ophthalmology, Tokyo Dental College, Ichikawa General Hospital, Chiba,

Japan

E-mail address of the corresponding author

E-mail: takamed@gmail.com

S Table 1 Correlations between best corrected visual acuity (BCVA) at 12 months and aberration factors.

	R	*P*-value
**Anterior HOAs (6 mm)**	0.1	0.590
**Posterior HOAs (6 mm)**	0.097	0.611
**Total HOAs (6 mm)**	0.014	0.940
**Anterior Comas (6 mm)**	0.204	0.973
**Posterior Comas (6 mm)**	0.120	0.530
**Total Comas (6 mm)**	0.117	0.537
**Anterior SAs (6 mm)**	0.342	0.065
**Posterior SAs (6 mm)**	0.019	0.920
**Total SAs (6 mm)**	0.297	0.111

Final visual acuity showed no correlation with the aberration factors.

HOAs, higher-order aberrations; Comas, coma aberrations; SAs, spherical aberrations

The data were analyzed using Spearman's correlation.
