# Supplementary material for: Optical characteristics after Descemet membrane endothelial keratoplasty: 1-year results
Source: PLoS One. 2020 Oct 14;15(10):e0240458. doi: 10.1371/journal.pone.0240458 (PMC7556451; doi:10.1371/journal.pone.0240458)
Supplement: Attachment — Submitted filename: PLOS DMEK 1-year review comments.docx [file pone.0240458.s004.docx]

PLOS ONE: Optical characteristics after DMEK: 1-year results

The authors provide excellent information about corneal characteristics throughout the first post-operative year after DMEK. Most of the suggestions below are minor. However, the entire Discussion section needs to be re-worked to provide a more cohesive analysis and discussion of limitations.

Abstract

- Line 45: would change “corneal shape” to “corneal characteristics”

- Methods need to make it clear at what time points patients were evaluated. “Patients who underwent DMEK at Yokohama Minami Kyosai Hospital were included and evaluated pre-operatively and at post-operative months 1, 3, 6, and 12 and compared to healthy controls. Corneal characteristics evaluated included (…)”

- Line 52: “Thirty eyes” should be “30 eyes”

- Line 61: change to “Despite achieving”

- Line 62: change to “showed a steeper posterior KV and higher corneal HOA’s”

Introduction

- Line 67: Dr. Eduard Zirm

- Prevalence of Fuchs dystrophy needs context (in the U.S.? Japan? Worldwide?)

- Would re-write first two paragraphs to focus more on differences of DSAEK vs. DMEK, as opposed to differences of PKP vs. DMEK

- Lines 86-88: Would delete this sentence as it is an overly broad statement that irregularities in topography correlate with vision. Would keep the focus on studies examining irregularities in post-DMEK eyes

- Lines 89-90: Delete or incorporate into previous paragraph about prior studies on DMEK outcomes

Materials and Methods

- Study design: how were phakic patients with Fuchs handled? Phakic DMEK, staged, triples?

- Study design: were patients with sub-epithelial scarring from chronic/severe Fuchs included?

- Later in the paper you state that all eyes undergoing DMEK had epithelial changes such as bullae or microcystic edema. Does this imply that eyes with visually significant guttae were not candidates for DMEK?

- Line 110: define “properly-sized graft”

- Line 112: what % size bubble was left?

- Line 120: “were evaluated preoperatively and at 1, 3, 6, and 12 months post-operatively”

- Line 127: delete “pre-op and 1, 3, 6, 12 months post-op” (redundant with earlier part of paragraph)

- Lines 127-129: delete “Since DMEK eyes may occasionally show central thinning”. Would address this in the Discussion section instead

- Line 129-130: “healthy eyes with no history of ocular disease or surgery with the exception of cataracts”

- Line 137: you list both “gender” and “sex”

Results

- Sample size: this paragraph is confusing. Power calculations should be done BEFORE patient recruitment based on expected differences in primary outcome. How can you do a power calculation after-the-fact based on post-op year #1 HOA data?

- Line 157: delete the word “current”

- Line 161: “All eyes showed epithelial disorders, such as bullae and microcystic changes” – Does this imply that DMEK was not performed for visually significant guttae? In this case, your population of patients undergoing DMEK may have more severe disease with implications for post-op remodeling, etc.

Table 1

- Change “numbers of eyes” to “number of eyes”

- Sex and eye: can just list % male and % right

- Add data on ethnicity

Results – VA and corneal thickness

- I would suggest rearranging the presentation of data as follows:

“BCVA after DMEK significantly improved throughout the post-operative period… However BCVA at all time points was still inferior to that of normal controls.

CCT after DMEK significantly decreased throughout the post-operative period…. And by post-op month 12 was comparable to normal controls.

PCT after DMEK significantly decreased throughout the post-operative period… However PCT never normalized as compared to healthy controls”

Table 2

- Need to re-arrange columns/rows as follows:

|  | Control eyes | DMEK eyes | P-value |
| --- | --- | --- | --- |
| BCVA |  | Pre-op: |  |
|  |  | POM #1: |  |
|  |  | POM #3: |  |
|  |  | POM #6: |  |
|  |  | POM #12: |  |

Keratometric values

- I would suggest rearranging the presentation of data as follows:

“Eyes with FECD had comparable anterior and total KV compared to healthy controls and these values did not change significantly after DMEK.

Eyes with FECD had steeper posterior KV compared to healthy controls. DMEK surgery resulted in a slight decrease in posterior KV but did not normalize value compared to healthy controls”

Lines 189-191: Unclear what you mean by “The anterior curvature became flat temporally, followed by a steep change. The posterior curvature showed a steep change temporally and flattened 1 month post-operatively”. How was this measured quantitatively? Did this occur in all post-DMEK eyes?

Discussion

- This whole section needs work

Summary paragraph suggestion:

“This study prospectively examined visual outcomes and corneal characteristics in eyes undergoing DMEK throughout the first post-operative year. After DMEK, all eyes experienced an improvement in BCVA and a reduction in HOAs, however none reached a level that was comparable to healthy controls. CCT and PCT both decreased after DMEK, with CCT normalizing but PCT remaining elevated compared to controls. Finally, eyes with FECD had steeper posterior KV compared to healthy controls and this value did not normalize after DMEK.”

Possible mechanisms explaining above findings:

- Replacement of central endothelial cells but low cell density peripherally

- Posterior stroma is more easily hydrated compared to anterior stroma, thus edema due to FECD is more likely to affect the posterior KV

- Would delete the Inoue et al reference unless you want to expand on the mechanism further – do you believe that the anatomy of the corneal nerves affects the location of corneal edema?

Limitations:

- Line 311: “relatively small number of Asian participants” – weren’t all the patients recruited from a Japanese hospital? You need to report ethnicity in Table 1

- Lines 312-314: this is not a limitation. Discussion of statistical power goes in the Methods section

- Lines 320-326: disagree with the rationale that you could not detect a correlation between HOAs and BCVA due to small intra-individual differences. If you were able to detect a statistically significant change in HOAs and BCVA at the various post-operative visits, then you should be able to calculate a correlation coefficient

- Line 326-327: would delete this sentence on DMEK vs DSAEK as it is not discussed anywhere else in the paper

- Would add the limitation that only eyes with epithelial changes (bullae or microcystic edema) were candidates for DMEK. Thus, your population of patients undergoing DMEK may have more severe disease compared to those studied in other papers (i.e. patients with guttae only)

- Would add limitation that even though some of your findings were statistically significant, there is a question about whether this translates to clinical significance (ex. a difference in posterior KV of -6.4 vs. -6.9)

Figure 2:

- Line 285 states that DMEK eyes show corneal thinning, however the corresponding image (Figure 2b) shows higher pachy values than Figure 2a

- Line 286 states that DMEK shows irregular posterior surface – I don’t think the resolution of the attached AS-OCT image supports that claim
