# Supplementary material for: Optical characteristics after Descemet membrane endothelial keratoplasty: 1-year results
Source: PLoS One. 2020 Oct 14;15(10):e0240458. doi: 10.1371/journal.pone.0240458 (PMC7556451; doi:10.1371/journal.pone.0240458)
Supplement: Attachment — Submitted filename: REVIEW_PONE-D-20-11108.docx [file pone.0240458.s005.docx]

**REVIEW FOR PONE-D-20-11108**

### 1. Summary of the research and overall impression

### The main research question was to evaluate the corneal shape after DMEK over 12 months and compare it to a control group. Claims included that posterior keratometric values (KV) of DMEK eyes did not compare as favorable as anterior ones to the control, that higher order aberrations (HOAs) and peripheral corneal thickness stayed significantly higher for DMEK eyes at 12 months than the control. The conclusion of the study was that DMEK eyes showed higher HOAs and more corneal irregularity. The claims are considered significant for the specialty and previous important literature was referenced. The optical quality of the cornea after DMEK was evaluated in two previous studies (1. [Optical quality of the cornea after Descemet membrane endothelial keratoplasty.](https://www.ncbi.nlm.nih.gov/pubmed/24784873) van Dijk K, Droutsas K, Hou J, Sangsari S, Liarakos VS, Melles GR Am J Ophthalmol. 2014 Jul;158(1):71-79.; 2. [Corneal higher-order aberrations after Descemet's membrane endothelial keratoplasty.](https://www.ncbi.nlm.nih.gov/pubmed/22197439) Ophthalmology. 2012 Mar;119(3):528-35. Rudolph M, Laaser K, Bachmann BO, Cursiefen C, Epstein D, Kruse FE). The current study fits into the literature concerning changes in HOA. Backscatter was not a parameter of this study.

The strengths of the manuscript are the postoperative follow-up over 12 months, the evaluation of relevant parameters (best-corrected visual acuity (BCVA), central corneal thickness, KV and HOA) and the number of eyes evaluated.

In order for the manuscript to proceed the authors should define the eyes receiving DMEK more precisely. The following questions should be addressed: “Were Triple-DMEKs included or excluded?“, “Was DMEK performed on only phakic, only pseudo-phakic or a mixed group of eyes?“. Another essential point for this manuscript is data collection and data interpretation for the parameter peripheral corneal thickness (PPT). More technical details should be added and clarification given. Otherwise one might have the impression that the authors missed something crucial. Up to my knowledge the company *CorneaGen*, having supplied the study center with DMEK tissues, sells grafts with the following diameters: 7.5 mm, 7.75 mm and 8.0 mm. Unfortunately, the used graft diameter was not explicitly stated. The reader might assume that it is equal to the descemetorhexis diameter of 8.0 mm. The PCT was measured at 9.0 mm. The difference should clearly be mentioned and consolidation given on data interpretation in the discussion section. Pictorial data should be chosen to give the reader an impression of the aforementioned technical details (edge of graft, point of measurement of PCT, diameter of standard graft used).

I consider this study to have sufficient potential and encourage the authors to re-submit a revised version. My overall recommendation is „Accept Manuscript after Revision“.

### 2. Discussion of specific areas for improvement

#### Major issues

The following essential points for this study should be addressed to let the manuscript proceed:

1. Define eyes receiving DMEK (as this might affect BCVA and/ or posterior corneal surface): Phakic, pseudo-phakic or mixed;

2. State the graft diameters used and discuss this in relation to the point of measurement of PCT. You might keep your data as it is but might want to explain that corneal edema is improving beyond the edge of the graft to some extent;

3. Depending on the used diameters data interpretation of PCT needs to be discussed;

4. Slit-lamp examination results might be addressed to mention corneal haze/ scarring (line 317-319) or other pathologies which may be present limiting BCVA;

5. The authors should clarify the discussion to avoid confusion (e.g. page 23, line 255-257: might this be caused by the difference between the edge of the graft and the 9-mm point of measurement of the PCT? ; page 23, line 258-259: the authors should specify the inconsistent findings; page 23, line 262-264: why inhomogeneous as this is a graft from a healthy donor cornea?; page 25, line 298-300: PCT affecting central BCVA?; page 25, line 306-308: explain “irregular distribution of the center-biased endothelium”);

6. Sample size and patient characteristics might be part of the Material and Methods section (page 9, line 146-162);

7. The authors state that cataract controls were used but report in their dataset for all 31 controls a logMAR-value between -0.08 to 0.0 (page 9, line 152 and *dataset table* [group “healthy control”; parameter BSCVA]). Clarification for this crucial point is needed.

#### Minor issues

Additional things to address to improve the overall manuscript whilst not affecting the conclusions:

1. Fix the uneven distribution of the tables over the word documents;

2. Please use the same abbreviations throughout the manuscript including tables. Keep in mind where to put the abbreviations in tables and avoid not abbreviating at all including supplemental material;

3. Use pictorial data which better matches the statements or simply improved illustrations (e.g.: Fig. 2b: the “inhomogeneous distribution” of endothelial cells is not clearly seen, Fig. 2c: peripheral edema is blurred or out-of-focus, Fig. 3a-d: irregular surfaces – whether anterior or posterior – cannot be seen clearly in either cross section). The authors might want to use a higher magnification, depict millimeter units to scale and identify the point of PCT measurement in relation to the edge of the graft;

4. Please avoid misplacing figure captions into main manuscript text sections. This is easy to avoid and makes a good impression for review;

5. Tables: Data seems to be missing for the control eyes at month 1, month 6 and month 12 while giving p-values for the respective follow-ups. The authors might adjust the layout to avoid confusion. N may be added for number of females/ males and number of eyes.

### 3. Any other points

# The authors made a great effort in data collection (number of parameters collected over 12 months of follow-up), although I am quite amazed that no data points are missing. Original data is made available and sufficient information is given to reproduce the study. Non-specialists are able to comprehend the idea behind this study. The conclusions do not overreach. Limitations of the study are discussed. Advance in the field is judged not applicable. I have no concerns about ethics or plagiarism but a professional statistician should evaluate the numbers/ data. I might be available for PLOSONE to look at a revised version of the manuscript.
