# Supplementary material for: Optical characteristics after Descemet membrane endothelial keratoplasty: 1-year results
Source: PLoS One. 2020 Oct 14;15(10):e0240458. doi: 10.1371/journal.pone.0240458 (PMC7556451; doi:10.1371/journal.pone.0240458)
Supplement: Attachment — Submitted filename: Plos_one_HOA_response_to_reviewers.docx [file pone.0240458.s006.docx]

*Reviewer #1: General Comments*

*Concise manuscript that discusses the effects of DMEK on corneal curvature and HOAs. However, the methodology requires some clarification before this manuscript can be considered for publication. The main issue is the criteria and justification for the control group. Moreover, extensive editing for grammar and English is required throughout the manuscript.*

- **Thank you very much for your review of our work. Your comments have aided us in significantly improving our manuscript. We have re-verified grammar and English of our manuscript which has been edited by a native English speaker. (**[**https://www.editage.jp/**](https://www.editage.jp/)**).**

*Specific Comments*

*Methods: Please state the inclusion criteria for DMEK cases e.g. FED or PBK etc. and inclusion for criteria for ‘age-matched’ controls.*

- **Our inclusion criteria were as follows: pseudophakic eyes without stromal scarring, but with stromal edema were included. Therefore, this study included 9 FED and 21 PBK, as shown in Table 1 (We did not find any differences in patients’ demographics, postop visual acuity and corneal HOAs between FED and PBK). Phakic DMEK or triple DMEK (combined with cataract surgery) were excluded. Fuchs keratopathy without corneal edema, but with corneal guttae not included in the study. Age-matched phakic eyes were selected as healthy controls.**
- **Following your suggestion, we have added the description as follows:**
- **Our inclusion criteria were pseudophakic eyes without stromal scarring, but with stromal edema. Phakic DMEK or triple DMEK (combined with cataract surgery) were excluded because of the difference in procedure. (Line 106–108)**

**Age-matched phakic eyes without history of ocular surgery or ocular surface disease were selected as healthy controls. (Line 111–112)**

*Methods: Why was 31 controls chosen instead of 30? What was definition of ‘healthy’ and what were criteria e.g. pseudophakic or phakic, myopic or refraction status etc. as these affect the corneal curvature.*

- **Thank you for these questions. The number of DMEK patients from July 2017 to Mar 2018 was 31; therefore we selected 31 phakic controls. However, one of DMEK patients dropped out from our study protocol, and this is why we analyzed 30 DMEK and 31 healthy controls. We have added the following description of healthy eyes:**

**“Age-matched phakic eyes without history of ocular surgery or ocular surface disease were selected concurrently as healthy controls.” (Line 111–112)**

*Methods: For sample size, please explain what is ‘cataract controls’*

- **Thank you for pointing this out. Unfortunately, this is a mistake. We have revised it to "phakic controls" as follows:**
- **Thirty-one phakic controls were obtained and compared with DMEK patients during the same period. (Line 157-158)**

*Methods: Please describe how the aberrations were derived from ASOCT with matching figure of the output and how the machine derives the values. This is important as each system varies on how the values are derived.*

- **Thank you for this comment. As shown in our previous paper, aberrations can be easily calculated by ASOCT (CASIA):**

**Sixteen rotating AS-OCT scans were used to reconstruct three-dimensional models of the entire corneal structure. The Casia SS-1000 system corrected distortions in the AS-OCT images based on the refractive index of the anterior surface. A corneal specialist (TH) carefully checked all AS-OCT images to ensure that the surface digitalization recognized by the automated inbuilt software was correct. Zernike coefficients were calculated using Zernike analysis as previously reported. In brief, the anterior and posterior corneal surfaces were reconstructed as a three-dimensional model from the corneal height data. The anterior, posterior, and total corneal aberrations at diameters of 4.0 mm and 6.0 mm were calculated separately using the installed ray tracing software (version 5.1). The refractive indices of the cornea and aqueous humor were set to 1.376 and 1.336, respectively. The wavefront aberration was expanded with normalized Zernike polynomials up to the 8th order. HOA was defined as the root mean square (RMS) of the 3rd to 8th order Zernike coefficients as follows:**

**SA was defined as the RMS of Z40 (spherical aberration) and Z60 (secondary spherical aberration). Coma was defined as the RMS of Z3-1 and Z31.**

*Methods: Were all DMEK patients psuedophakic? Were all the controls psuedophakic? If so, please include the HOA reading for the controls before and after cataract surgery.*

- **Thank you for this question. All DMEK patients were pseudophakic, and healthy controls were phakic without cataract. We have revised the manuscript as follows:**

**“Our inclusion criteria were pseudophakic eyes without stromal scarring, but with stromal edema. Phakic DMEK or triple DMEK (combined with cataract surgery) were excluded because of the difference in procedure.” (Line 106–108)**

**“Age-matched phakic eyes without history of ocular surgery or ocular surface disease were selected concurrently as healthy controls” (Line 111–112)**

*Reviewer #2: The authors provide excellent information about corneal characteristics throughout the first post-operative year after DMEK. Most of the suggestions below are minor. However, the entire Discussion section needs to be re-worked to provide a more cohesive analysis and discussion of limitations.*

*Full comments have been uploaded as an attached document*

- **Thank you very much for your review of our work. Your encouraging comments have aided us in significantly improving our manuscript.**

*Abstract*

*- Line 45: would change “corneal shape” to “**corneal characteristics”*

- **Following your suggestion, we have revised the text as follows:**

**“To evaluate the corneal characteristics after Descemet membrane endothelial keratoplasty (DMEK) compared with normal corneas.” (Line 45–46)**

*- Methods need to make it clear at what time points patients were evaluated. “**Patients who underwent DMEK at Yokohama Minami Kyosai Hospital were included and evaluated pre-operatively and at post-operative months 1, 3, 6, and 12 and compared to healthy controls. Corneal characteristics evaluated included (…)”*

- **Following your suggestion, we have revised the manuscript as follows:**

**Patients who underwent DMEK at Yokohama Minami Kyosai Hospital were included and prospectively evaluated pre-operatively and at post-operative months 1, 3, 6, and 12, and compared to healthy controls. Corneal characteristics evaluated included corneal curvature (keratometric value [ KV]; D), central corneal thickness (CCT), peripheral corneal thickness (PCT), and corneal higher-order aberrations [HOAs] at 6.0 mm diameter, calculated by anterior segment optical coherence tomography and logarithm of the minimal angle of resolution [logMAR]. (Line 47–53)**

*- Line 52: “Thirty eyes” should be “30 eyes”*

- **Following your suggestion, we have revised the description as follows:**

**“A total of 30 eyes of 30 patients” (Line 54)**

*- Line 61: change to “**Despite achieving”*

**Following your suggestion, we have revised the description as follows:**

- **“Despite achieving good visual function and excellent corneal clarity, ” (Line 63)**

*- Line 62: change to “**showed a steeper posterior KV and higher corneal HOA’s”*

**Following your suggestion, we have revised the description as follows:**

- **“Despite achieving good visual function and excellent corneal clarity, eyes that underwent DMEK showed a steeper posterior KV and higher corneal HOAs than normal eyes even at 12 months after surgery.” (Line 63–65)**

*Introduction*

*- Line 67:* *Dr. Eduard Zirm*

- **Following your suggestion, we have revised the description as follows:**

**“Dr. Eduard Zirm” (Line 69)**

*- Prevalence of Fuchs dystrophy needs context (in the U.S.? Japan? Worldwide?)*

- **Thank you for pointing this out. We meant worldwide.**

**Following your suggestion, we have added the description as follows:**

**“In the worldwide population over the age of 40 years, the incidence of Fuchs endothelial corneal dystrophy (FECD), which requires corneal transplantation, is approximately 5%” (Line 71–73)**

*- Would re-write first two paragraphs to focus more on differences of DSAEK vs. DMEK, as opposed to differences of PKP vs. DMEK*

- **Following your suggestion, we have revised the description as follows:**

**“**

**Therefore, endothelial keratoplasty (EK), which includes Descemet’s stripping automated endothelial keratoplasty (DSAEK] and Descemet’s membrane endothelial keratoplasty (DMEK), has been introduced worldwide and found to have advantages over PKP in terms of astigmatism, risk of graft rejection, and visual recovery [4-6].**

**Especially, the minimally invasive surgical procedure DMEK, developed by Dr. Melles in 2006 [7], involves the selective exchange of a complex of Descemet’s membrane and the endothelial layer. DMEK offers two primary advantages: 1) rapid visual recovery with a better final visual outcome than other keratoplasty techniques [8, 9], and 2) an extremely low incidence of graft rejection even when compared to DSAEK [10-12]. Previous studies have proved the superiority of DMEK to either DSAEK or ultra-thin DSAEK in terms of visual function [13-17].” (Line 77–87)**

*- Lines 86-88: Would delete this sentence as it is an overly broad statement that irregularities in topography correlate with vision. Would keep the focus on studies examining irregularities in post-DMEK eyes*

- **Following your suggestion, we have deleted the following description:**

**“We have already reported that topographic changes and improvements in irregularity after keratoplasty or corneal disease show a strong correlation with visual function [13, 15-21].**

**As we have already shown, the visual acuity may occasionally not reach 20/20. Moreover, irregular topographic patterns may appear even after DMEK.”**

*- Lines 89-90: Delete or incorporate into previous paragraph about prior studies on DMEK outcomes*

- **Following your suggestion, we have deleted the following description:**

**“As we have already shown, the visual acuity may occasionally not reach 20/20. Moreover, irregular topographic patterns may appear even after DMEK. “**

*Materials and Methods*

*- Study design: how were phakic patients with Fuchs handled? Phakic DMEK, staged, triples?*

**Our inclusion criteria were as follows: pseudophakic eyes without stromal scarring, but with stromal edema. Phakic DMEK or triple DMEK (combined with cataract surgery) were excluded because of the difference in procedure.**

*- Study design: were patients with sub-epithelial scarring from chronic/severe Fuchs included?*

**Our inclusion criteria were as follows: pseudophakic eyes without stromal scarring, but with stromal edema. Phakic DMEK or triple DMEK (combined with cataract surgery) were excluded because of the difference in procedure.**

*- Later in the paper you state that all eyes undergoing DMEK had epithelial changes such as bullae or microcystic edema. Does this imply that eyes with visually significant guttae were not candidates for DMEK?*

- **Our inclusion criteria were as follows: pseudophakic eyes without stromal scarring, but with stromal edema. Phakic DMEK or triple DMEK (combined with cataract surgery) were not included. Fuchs keratopathy without corneal edema, but with corneal guttae was not included in our study.**

**Following your suggestion, we have revised the text as below;**

**“Our inclusion criteria were pseudophakic eyes without stromal scarring, but with stromal edema. Phakic DMEK or triple DMEK (combined with cataract surgery) were excluded because of the difference in procedure.” (Line 106–108)**

*- Line 110: define “properly-sized graft”*

- **Thank you for pointing this out.**

**We always use a donor punch with a diameter of 7.75 mm, 8.0 mm, or 8.25 mm, and try to make the area of descemetorhexis at least 9.0 mm. However, the size of the descemetorhexis graft can change depending on the corneal curvature of the donor or host.**

**Following your suggestion, we have revised the description as follows:**

**“Briefly, after the creation of a descemetorhexis with an approximate diameter of 9.0-mm under air, an appropriately-sized graft (7.75 mm, 8.0 mm, or 8.25 mm) was inserted using an intraocular lens inserter (WJ-60M®; Santen, Osaka, Japan)” (Line 116–118)**

**In general, Descemet membrane was then gently peeled from the stroma to obtain a Descemet membrane/corneal endothelial cell flap that was 8 mm in diameter.**

*- Line 112: what % size bubble was left?*

**For fixation of the DMEK graft, the anterior chamber volume was filled with 20% sulfur hexafluoride (SF6) until 80% of the AC volume was filled.**

**Following your suggestion, we have revised the description as follows:**

**“Briefly, after the creation of a descemetorhexis with an approximate diameter of 9.0-mm under air, an appropriately-sized graft (7.75 mm, 8.0 mm, or 8.25 mm) was inserted using an intraocular lens inserter (WJ-60M®; Santen, Osaka, Japan), and was unfolded and fixated with 20% sulfur hexafluoride (SF6) until 80% of the anterior chamber volume was filled.” (Line 116–119)**

*- Line 120: “**were evaluated preoperatively and at 1, 3, 6, and 12 months post-operatively”*

**Following your suggestion, we have revised the description.**

*- Line 127: delete “pre-op and 1, 3, 6, 12 months post-op” (redundant with earlier part of paragraph)*

**Following your suggestion, we have deleted the description.**

*- Lines 127-129: delete “Since DMEK eyes may occasionally show central thinning”. Would address this in the Discussion section instead*

**Following your suggestion, we have moved the description to the Discussion.**

*- Line 129-130: “healthy eyes with no history of ocular disease or surgery with the exception of cataracts”*

**Following your suggestion, we have removed the description, and revised the text as follows:**

**“Age-matched phakic eyes without history of ocular surgery or ocular surface disease were selected concurrently as healthy controls” (Line 111–112)**

*- Line 137: you list both “gender” and “sex”*

- **Following your suggestion, we have removed “gender”. (Line 142)**

*Results*

*Sample size: this paragraph is confusing. Power calculations should be done BEFORE patient recruitment based on expected differences in primary outcome. How can you do a power calculation after-the-fact based on post-op year #1 HOA data?*

**Thank you for this question. We used our preliminary data (not published), and calculated the statistic power using these values. We are convinced that there was a similar tendency regarding the HOAs of DMEK eyes at one year and healthy controls.**

*- Line 157: delete the word “current”*

- **Following your suggestion, we have removed the word “current”. (Line 161)**

*- Line 161: “All eyes showed epithelial disorders, such as bullae and microcystic changes”*

*– Does this imply that DMEK was not performed for visually significant guttae? In this case, your population of patients undergoing DMEK may have more severe disease with implications for post-op remodeling, etc.*

- **Thank you for this question; we fully agree with your opinion.**

**Our inclusion criteria were as follows: pseudophakic eyes without stromal scarring, but with stromal edema. Phakic DMEK or triple DMEK (combined with cataract surgery) were not included. Fuchs keratopathy without corneal edema, but with corneal guttae was not included in our study.**

*Table 1*

*- Change “numbers of eyes” to “number of eyes”*

**Following your suggestion, we have revised the text to “number of eyes”.**

*- Sex and eye: can just list % male and % right*

**Following your suggestion, we have revised this for clarity.**

*- Add data on ethnicity*

**Following your suggestion, we have added data on ethnicity.**

*Results – VA and corneal thickness*

*- I would suggest rearranging the presentation of data as follows:*

*“**BCVA after DMEK significantly improved throughout the post-operative period… However BCVA at all time points was still inferior to that of normal controls.*

*CCT after DMEK significantly decreased throughout the post-operative period…. And by post-op month 12 was comparable to normal controls.*

*PCT after DMEK significantly decreased throughout the post-operative period… However, PCT never normalized as compared to healthy controls”*

- **We fully agree with you. Your suggestions were very valuable and useful to us.**

**Following your suggestions, we have revised the text as follows:**

**“Visual acuity and corneal thickness**

**BCVA significantly improved after DMEK throughout the post-operative period as follows: from 0.87 ± 0.52 preoperatively to 0.26 ± 0.23 at 1 month (Table 2, p < 0.001), 0.12 ± 0.15 at 3 months (p < 0.001), 0.05 ± 0.11 at 6 months (p < 0.001), and 0.04 ± 0.11 (p < 0.001) at 12 months postoperatively. However, BCVA was still inferior to that of normal controls at all time points.**

**CCT after DMEK significantly decreased throughout the post-operative period up to 6 months after surgery; at 12 months after surgery it was comparable to normal controls. CCT significantly decreased from 682 ± 100 μm preoperatively to 523 ± 49 μm (p < 0.001), 508 ± 38 μm (p < 0.001), 512 ± 38 μm (p < 0.001) and 518 ± 35 μm (p < 0.001) at 1, 3, 6, and 12 months postoperatively, respectively. CCT was significantly thinner in DMEK eyes than in control eyes (531 ± 32 μm) at 3 and 6 months postoperatively, whereas it was similar to that in control eyes 12 months postoperatively.**

**Although PCT after DMEK significantly decreased throughout the post-operative period, PCT never normalized as compared to healthy controls. Preoperative PCT was 821 ± 106 μm and changed to 765 ± 54 μm (p < 0.001), 723 ± 43 μm (p < 0.001), 710 ± 39 μm (p < 0.001), and 704 ± 41 μm (p = 0.002) at 1, 3, 6, and 12 months postoperatively, respectively. All PCTs were significantly larger in DMEK eyes compared to those in control eyes (669 ± 38 μm) (p < 0.001).” (Line 171–188)**

*Table 2*

*- Need to re-arrange columns/rows as follows:*

*Control eyes	DMEK eyes	P-value*

*BCVA	Pre-op:*

*POM #1:*

*POM #3:*

*POM #6:*

*POM #12:*

- **We fully agree with you. Your suggestion was very valuable and useful to us.**

**Following your suggestion, we have revised the Table 2 to improve its layout.**

*Keratometric values*

*- I would suggest rearranging the presentation of data as follows:*

*“Eyes after DMEK had comparable anterior and total KV compared to healthy controls and these values did not change significantly after DMEK.*

*Eyes after DMEK had steeper posterior KV compared to healthy controls. DMEK surgery resulted in a slight decrease in posterior KV but did not normalize value compared to healthy controls”*

- **We fully agree with you. Your suggestions were very valuable and useful to us.**

**Following your suggestion, we have revised the text as follows:**

**Keratometric values**

**Table 3 shows the time course of KV. Eyes after DMEK had comparable anterior and total KV compared to healthy controls and these values did not change significantly after DMEK. Anterior KV changed from 49.1 ± 2.0 D preoperatively to 49.0 ± 1.6 D at 1 month (p = 0.695), 49.0 ± 1.7 D at 3 months (p = 0.695), 49.4 ± 1.2 D at 6 months (p = 0.918), and 49.4 ± 1.6 D at 12 months (p = 0.853) postoperatively. Total KV changed from 42.8 ± 1.9 D preoperatively to 42.5 ± 1.5 D at 1 month (p = 0.679), 42.9 ± 1.4 D at 3 months (p = 0.679), 43.1 ± 1.0 D at 6 months (p = 0.679), and 43.1 ± 1.4 D at 12 months (p = 0.600) postoperatively.**

**Eyes after DMEK had steeper posterior KV compared to healthy controls. DMEK surgery resulted in a slight decrease in posterior KV, but the value did not normalize compared to healthy controls. Posterior KV changed from -6.6 ± 0.5 D preoperatively to -6.6 ± 0.4 D at 1 month (p = 0.589), -6.5 ± 0.3 D at 3 months (p = 0.589), -6.5 ± 0.3 D at 6 months (p = 0.395), and -6.4 ± 0.3 D at 12 months (p = 0.351) postoperatively. Although there were no significant differences in the anterior or total KVs, the posterior KV was larger in DMEK eyes than in control eyes (p < 0.05). (Line 201–215)**

*Lines 189-191: Unclear what you mean by “The anterior curvature became flat temporally, followed by a steep change. The posterior curvature showed a steep change temporally and flattened 1 month post-operatively”. How was this measured quantitatively? Did this occur in all post-DMEK eyes?*

- **Thank you for pointing this out. We agree with you that the sentence is confusing.**

**We have, therefore, removed the sentence.**

*Discussion*

*- This whole section needs work*

*Summary paragraph suggestion:*

*“This study prospectively examined visual outcomes and corneal characteristics in eyes　undergoing DMEK throughout the first post-operative year. After DMEK, all eyes experienced an improvement in BCVA and a reduction in HOAs, however none reached a level that was comparable to healthy controls. CCT and PCT both decreased after DMEK, with CCT normalizing but PCT remaining elevated compared to controls. Finally, eyes with FECD had steeper posterior KV compared to healthy controls and this value did not normalize after DMEK.”*

- **Thank you for this kind suggestion. Following your suggestion, we have revised the first paragraph of the discussion as follows:**

**“This study prospectively examined visual outcomes and corneal characteristics in eyes undergoing DMEK throughout the first post-operative year. After DMEK, all eyes experienced an improvement in BCVA and a reduction in HOAs; however, none of them reached a level that was comparable to healthy controls. Both central and peripheral corneal thickness (CCT and PCT) decreased after DMEK, with CCT normalizing but PCT remaining thicker compared to controls. Eyes after DMEK had steeper posterior KV compared to healthy controls and this value did not normalize even at 12 months after surgery.” (Line 273-279)**

*Possible mechanisms explaining above findings:*

*-* *Replacement of central endothelial cells but low cell density peripherally*

*-* *Posterior stroma is more easily hydrated compared to anterior stroma, thus edema due to FECD is more likely to affect the posterior KV*

- **We fully agree with your suggestions. We have revised the discussion according to these suggestions. (Line 272–297)**

*- Would delete the Inoue et al reference unless you want to expand on the mechanism further – do you believe that the anatomy of the corneal nerves affects the location of corneal edema?*

- **We fully agree with your suggestion. Following your suggestion, we have deleted the relevant text and citation from the discussion. (Line 272–297)**

*Limitations:*

*- Line 311: “relatively small number of Asian participants” – weren’t all the patients recruited from a Japanese hospital? You need to report ethnicity in Table 1*

- **Thank you for pointing this out. We have revised the text to clarify that all patients were Japanese ethnicity.**

*- Lines 312-314: this is not a limitation. Discussion of statistical power goes in the Methods section*

- **Thank you for pointing this out. We agree that this part is not necessary in the discussion section.**

**Following your suggestion, this text was removed. We have briefly mentioned that statistical power calculation and appropriate sample size is a strength of the study.**

*- Lines 320-326: disagree with the rationale that you could not detect a correlation between HOAs and BCVA due to small intra-individual differences. If you were able to detect a statistically significant change in HOAs and BCVA at the various post-operative visits, then you should be able to calculate a correlation coefficient*

- **We fully agree with you. Following your suggestion, we have deleted the discussion regarding DMEK vs DSAEK.**

*- Line 326-327: would delete this sentence on DMEK vs DSAEK as it is not discussed anywhere else in the paper*

- **We fully agree with you. Following your suggestion, we have deleted the discussion regarding DMEK vs DSAEK.**

*- Would add the limitation that* *only eyes with epithelial changes (bullae or microcystic edema) were candidates for DMEK. Thus, your population of patients undergoing DMEK may have more severe disease compared to those studied in other papers (i.e. patients with guttae only)*

*- Would add limitation that* *even though some of your findings were statistically significant, there is a question about whether this translates to clinical significance (ex. a difference in posterior KV of -6.4 vs. -6.9)*

**We fully agree with you. Following your suggestion, we have revised the discussion as follows:**

**“This study had some strengths and limitations. The strengths include that we calculated the statistical power and used the appropriate sample size. Furthermore, our prospective study had no missing data. The study also had the following limitations. First, only Asian eyes (Japanese) with epithelial changes suggestive of more severe disease, such as bullae or microcystic edema, were included. Second, the clinical significance of some of our findings remains unknown, despite their statistical significance. Third, backscatter and corneal haze were not evaluated because AS-OCT is not equipped for backscatter or densitometry.” (Line 333–339)**

*Figure 2:*

*- Line 285 states that DMEK eyes show corneal thinning, however the corresponding image (Figure 2b) shows higher pachy values than Figure 2a*

*- Line 286 states that DMEK shows irregular posterior surface – I don’t think the resolution of the attached AS-OCT image supports that claim*

- **Thank you for pointing this out. Following your suggestion, we have revised Figure 2.**

*Reviewer #3: REVIEW FOR PONE-D-20-11108*

*1. Summary of the research and overall impression*

*The main research question was to evaluate the corneal shape after DMEK over 12 months and compare it to a control group. Claims included that posterior keratometric values (KV) of DMEK eyes did not compare as favorable as anterior ones to the control, that higher order aberrations (HOAs) and peripheral corneal thickness stayed significantly higher for DMEK eyes at 12 months than the control. The conclusion of the study was that DMEK eyes showed higher HOAs and more corneal irregularity. The claims are considered significant for the specialty and previous important literature was referenced. The optical quality of the cornea after DMEK was evaluated in two previous studies (1. Optical quality of the cornea after Descemet membrane endothelial keratoplasty. van Dijk K, Droutsas K, Hou J, Sangsari S, Liarakos VS, Melles GR Am J Ophthalmol. 2014 Jul;158(1):71-79.; 2. Corneal higher-order aberrations after Descemet's membrane endothelial keratoplasty. Ophthalmology. 2012 Mar;119(3):528-35. Rudolph M, Laaser K, Bachmann BO, Cursiefen C, Epstein D, Kruse FE). The current study fits into the literature concerning changes in HOA. Backscatter was not a parameter of this study.*

*The strengths of the manuscript are the postoperative follow-up over 12 months, the evaluation of relevant parameters (best-corrected visual acuity (BCVA), central corneal thickness, KV and HOA) and the number of eyes evaluated.*

*In order for the manuscript to proceed the authors should define the eyes receiving DMEK more precisely. The following questions should be addressed: “Were Triple-DMEKs included or excluded?“, “Was DMEK performed on only phakic, only pseudo-phakic or a mixed group of eyes?“. Another essential point for this manuscript is data collection and data interpretation for the parameter peripheral corneal thickness (PPT). More technical details should be added and clarification given. Otherwise one might have the impression that the authors missed something crucial. Up to my knowledge the company CorneaGen, having supplied the study center with DMEK tissues, sells grafts with the following diameters: 7.5 mm, 7.75 mm and 8.0 mm. Unfortunately, the used graft diameter was not explicitly stated. The reader might assume that it is equal to the descemetorhexis diameter of 8.0 mm. The PCT was measured at 9.0 mm. The difference should clearly be mentioned and consolidation given on data interpretation in the discussion section.* *Pictorial data should be chosen to give the reader an impression of the aforementioned technical details (edge of graft, point of measurement of PCT, diameter of standard graft used).*

*I consider this study to have sufficient potential and encourage the authors to re-submit a revised version. My overall recommendation is „Accept Manuscript after Revision“.*

- **Thank you very much for your review of our work. Your comments have aided us in significantly improving our manuscript. We have clarified the inclusion/exclusion criteria following the reviewer comments.**

*2. Discussion of specific areas for improvement*

*Major issues*

*The following essential points for this study should be addressed to let the manuscript proceed:*

1. *Define eyes receiving DMEK (as this might affect BCVA and/ or posterior corneal surface): Phakic, pseudo-phakic or mixed;*

- **Our inclusion criteria were as follows: pseudophakic eyes without stromal scarring, but with stromal edema. Phakic DMEK or triple DMEK (combined with cataract surgery) were excluded. Fuchs keratopathy without corneal edema, but with corneal guttae was not included in our study. Age-matched phakic eyes were selected as healthy controls.**
- **Following your suggestion, we have added the description as follows:**

**“Our inclusion criteria were pseudophakic eyes without stromal scarring, but with stromal edema. Phakic DMEK or triple DMEK (combined with cataract surgery) were excluded because of the difference in procedure.” (Line 106–108)**

*2. State the graft diameters used and discuss this in relation to the point of measurement of PCT. You might keep your data as it is but might want to explain that corneal edema is improving beyond the edge of the graft to some extent;*

- **We fully agree with you. We always use a donor punch with a diameter of 7.75mm, 8.0mm, or 8.25mm, and try to make the area of descemetorhexis at least 9.0 mm. However, the size of the descemetorhexis graft can change depending on the corneal curvature of donor or host.**

**Following your suggestion, we have revised the text as below:**

**“Briefly, after the creation of a descemetorhexis with an approximate diameter of 9.0-mm under air, an appropriately-sized graft (7.75 mm, 8.0 mm, or 8.25 mm) was inserted using an intraocular lens inserter (WJ-60M®; Santen, Osaka, Japan).” (Line 116–118)**

**In general, Descemet membrane was then gently peeled from the stroma at approximately 9 mm to obtain a Descemet membrane/corneal endothelial cell flap that was 8 mm in diameter.**

**Further, we have revised the Figure 3 to make it easier for readers to understand the manuscript.**

2. *Depending on the used diameters data interpretation of PCT needs to be discussed;*

- **We always use a donor punch with a diameter of 7.75mm, 8.0mm, or 8.25mm, and try to make the area of descemetorhexis at least 9.0 mm. However, the size of the descemetorhexis graft can change depending on the corneal curvature of donor or host.**

**We have added the following text to the manuscript:**

**As DMEK replaces only the central area of the back-layer of the cornea, it is possible that this results in high ECD in the central area, and low ECD in the peripheral area. Moreover, since the posterior stroma is more easily hydrated compared to the anterior stroma, the improvement of corneal edema after DMEK is more likely to affect the posterior KV than the anterior KV. (Line 281–285)**

**Further, we have revised the Figure 3 to make it easier for readers to understand the manuscript.**

3. *Slit-lamp examination results might be addressed to mention corneal haze/ scarring (line 317-319) or other pathologies which may be present limiting BCVA;*

- **Following your suggestion, we have added the following text to the manuscript:**

**“Our inclusion criteria were pseudophakic eyes without stromal scarring, but with stromal edema. Phakic DMEK or triple DMEK (combined with cataract surgery) were excluded because of the difference in procedure.” (Line 106–109)**

4. *The authors should clarify the discussion to avoid confusion (e.g. page 23, line 255-257: might this be caused by the difference between the edge of the graft and the 9-mm point of measurement of the PCT? ; page 23, line 258-259: the authors should specify the inconsistent findings; page 23, line 262-264: why inhomogeneous as this is a graft from a healthy donor cornea?; page 25, line 298-300: PCT affecting central BCVA?; page 25, line 306-308: explain “irregular distribution of the center-biased endothelium”);*

- **Thank you for these suggestions. As you suggested, the discussion was confusing, and we have revised the discussion as follows:**

**“This study prospectively examined visual outcomes and corneal characteristics in eyes undergoing DMEK throughout the first post-operative year. After DMEK, all eyes experienced an improvement in BCVA and a reduction in HOAs; however, none of them reached a level that was comparable to healthy controls. Both central and peripheral corneal thickness (CCT and PCT) decreased after DMEK, with CCT normalizing but PCT remaining thicker compared to controls. Eyes after DMEK had steeper posterior KV compared to healthy controls and this value did not normalize even at 12 months after surgery.**

**A thorough consideration of the corneal curvature is important to explain these findings. As DMEK replaces only the central area of the back-layer of the cornea, it is possible that this results in high ECD in the central area, and low ECD in the peripheral area. Moreover, since the posterior stroma is more easily hydrated compared to the anterior stroma, the improvement of corneal edema after DMEK is more likely to affect the posterior KV than the anterior KV.” (Line 273–285)**

*6. Sample size and patient characteristics might be part of the Material and Methods section (page 9, line 146-162);*

- **Thank you for pointing this out. We agree that text regarding how to calculate statistic power, or how to include patients should be placed in the Methods, but how many patients were obtained after calculation, or observation should be placed in the Results.**

*7. The authors state that cataract controls were used but report in their dataset for all 31 controls a logMAR-value between -0.08 to 0.0 (page 9, line 152 and dataset table [group “healthy control”; parameter BSCVA]). Clarification for this crucial point is needed.*

- **Thank you for pointing out this mistake. We have revised it to "phakic controls" as follows:**

**“Thirty-one phakic controls were obtained and compared with DMEK patients during the same period.” (Line 157–158)**

*Minor issues*

*Additional things to address to improve the overall manuscript whilst not affecting the conclusions:*

1. *Fix the uneven distribution of the tables over the word documents;*

- **Thank you for pointing this out. Following your suggestion, we have revised the Tables.**

2. *Please use the same abbreviations throughout the manuscript including tables. Keep in mind where to put the abbreviations in tables and avoid not abbreviating at all including supplemental material;*

- **We fully agree with your suggestion. Following your suggestion, we have revised the abbreviations in the manuscript.**

*3. Use pictorial data which better matches the statements or simply improved illustrations (e.g.: Fig. 2b: the “inhomogeneous distribution” of endothelial cells is not clearly seen, Fig. 2c: peripheral edema is blurred or out-of-focus, Fig. 3a-d: irregular surfaces – whether anterior or posterior – cannot be seen clearly in either cross section). The authors might want to use a higher magnification, depict millimeter units to scale and identify the point of PCT measurement in relation to the edge of the graft;*

- **We fully agree with you. Following your suggestion, we have revised Figures 2 and 3.**

3. *Please avoid misplacing figure captions into main manuscript text sections. This is easy to avoid and makes a good impression for review;*

- **We fully agree with you. Following your suggestion, we have revised the Figure captions so that they are inserted after the first mention of the figure in the text, as per the journal guidelines.**

4. *Tables: Data seems to be missing for the control eyes at month 1, month 6 and month 12 while giving p-values for the respective follow-ups. The authors might adjust the layout to avoid confusion. N may be added for number of females/ males and number of eyes.*

- **Since controls are control eyes (no surgery), there is no postoperative data. Following the reviewers’ suggestion, we have revised the layout of the table.**
- **Your suggestions were very valuable and useful to us.**

**Following your suggestion, we have revised Table 2.**

*3. Any other points*

*The authors made a great effort in data collection (number of parameters collected over 12 months of follow-up), although I am quite amazed that no data points are missing. Original data is made available and sufficient information is given to reproduce the study. Non-specialists are able to comprehend the idea behind this study. The conclusions do not overreach. Limitations of the study are discussed. Advance in the field is judged not applicable. I have no concerns about ethics or plagiarism but a professional statistician should evaluate the numbers/ data. I might be available for PLOSONE to look at a revised version of the manuscript.*

- **Thank you for these comments. Regarding statistics, prof. Hidenori Takahashi has a qualification in statistics.**
