# Supplementary material for: Optical characteristics after Descemet membrane endothelial keratoplasty: 1-year results
Source: PLoS One. 2020 Oct 14;15(10):e0240458. doi: 10.1371/journal.pone.0240458 (PMC7556451; doi:10.1371/journal.pone.0240458)
Supplement: Attachment — Submitted filename: Response_to_Plos_one_DMEK_HOA_2nd_revision.docx [file pone.0240458.s007.docx]

**Reviewer #2: Thank you for all your thoughtful responses and modifications. The revised manuscript is much more comprehensive and coherent. Most of my comments below are minor; however, the Discussion section still needs to be significantly re-worked.**

- Thank you very much for your review of our work. Your comments have helped us to significantly improve our manuscript.

**Introduction**

**- Line 72: would modify, as not all patients with Fuchs requires corneal transplantation**

**- Would shorten paragraph about transition from PKP to EK**

- Following your suggestion, we have revised the description about Fuchs endothelial dystrophy and shortened paragraph about transition from PKP to EK. (Line 70-79)

**- Dr. Gerrit Melles**

- Following your suggestion, we have revised the description as follow.

‘Dr. Gerrit Melles’ (Line 80)

**Methods**

**- Line 117: modify to "****appropriately under-sized graft"**

- Following your suggestion, we have revised the description as follow.

‘appropriately under-sized graft’ (Line 115)

**Results**

**- Line 153-154: need to make clear that the data and calculations were based on non-published preliminary data**

- Following your suggestion, we have revised the description as follow.

‘From our calculations using unpublished preliminary data (the HOAs of DMEK eyes at one year and of control eyes were 0.97 ± 0.43 μm and 0.61 ± 0.19, respectively)’ (Line 151-152)

**- Patient characteristics: Would move "All patients were of Japanese ethnicity" to line 164, after discussion of differences in age/sex**

- Following your suggestion, we have moved the patient description after description of age and sex. (Line 162)

**- Line 213-215: Would delete, redundant with prior paragraphs**

- Following your suggestion, we have deleted the redundant description.

**- HOA's: After each paragraph, I would add a sentence comparing the DMEK outcomes to control eyes. Currently this comparison occurs in lines 260-264, but I think it would be easier for the reader if the comparison was done at the time each factor is discussed**

- Following your suggestion, we have replaced these descriptions. (Line 217-218, 243-244, and 253-255)

**Discussion**

**- This whole section needs to be reworked so that it's more organized and coherent**

**- A few specific points below:**

**- Lines 302-303: was peripheral edema detected even at POM #12? If so, in how many eyes did this occur?**

- There was no eye with epithelial disorder such as bullae, or cystic change at 12 months after DMEK. We have revised the description as follows.

‘At the early phase after DMEK [within 1 month], peripheral edema was occasionally detected in the peripheral cornea as bullae (c) or as an irregular epithelium (d).’ (Line 298-300)

**- Lines 319-320: "which coincided with normalization of PCT at 12 months" - but I thought that PCT never normalized compared to controls?**

- Following your suggestion, we have replaced the description.

‘It is noteworthy that corneal HOAs were reduced at 6 to 12 months after DMEK, which coincided with improvement of PCT at 12 months.’ (Line 319-321)

**- Lines 320-322: I don't think you can make this conclusion, especially since your analysis did not show correlation between any factor and VA**

- Following your suggestion, we have revised the description as follows.

‘Thus, we consider that the assessment of PCT would be a clinically relevant parameter when evaluating improvement in corneal edema after DMEK.’ (Line 321-322)

**- Another point that should be added is the relatively high % of patients with PBK versus Fuchs. Patients with PBK likely have decreased reserve of healthy peripheral endothelium compared to Fuchs, which may contribute to your finding that PCT does not normalize**

- Following your suggestion, we have added a theorical comment in the discussion as follows.

‘In our study, 21 of 30 DMEK cases (70%) were PBK. The high percentage of PBK in our cohort may contribute to the findings that PCT did not normalize after surgery.’ (Line 318-319)

**- Lines 333-334: Would delete sentence about statistical power**

- Following your suggestion, we have deleted the discussion about statistic power.

**Table 2**

**- Would reformat columns so that it matches that of Table 3**

- Following your suggestion, we have reformatted Table 2.

**Reviewer #3: REVIEW FOR PONE-D-20-11108**

**1. Overall impression**

**I still consider this study to have sufficient potential for publication. My overall recommendation is „Accept Manuscript after Revision“.**

**2. Open points**

**Cf. Revision 1:**

**State the graft diameters used and discuss this in relation to the point of measurement of PCT. You might keep your data as it is but might want to explain that corneal edema is improving beyond the edge of the graft to some extent;**

**Pictorial data should be chosen to give the reader an impression of the aforementioned technical details (edge of graft, point of measurement of PCT, diameter of standard graft used).**

**Unanswered or open points of major concern affecting the conclusions of the study/ concerning the methods used:**

**Unfortunately, the authors did not yet explicitly state that all measurements of the peripheral corneal thickness were at least 0.75-1.25 mm beyond the edge of the graft/ in diseased host cornea. Thus, the peripheral edema measured cannot be directly affected by DMEK surgery. This inevitably has effects on the interpretation of the data. Figure 2 does not show an inhomogenous distribution as stated in the manuscript text. The text should clearly refer to the addressed figure/ sub-figure.**

**The pictorial data needs to be adjusted accordingly. The cell density of the graft in figure 2 seems to be higher than of the healthy cornea. All points should be fixed in the schematic images. In addition, the used arrows and circle need to be explained to the reader.**

- Following your suggestion, we have revised the caption of Fig.2 and added the discussion as follows.
- ‘inhomogenous’ → ’typical’ (Line 277)

‘In comparison with a healthy cornea (a), DMEK eyes (b) contain few healthy endothelia in the peripheral cornea, because the graft size (around 8.0 mm) tends to be smaller than the stripping area (around 9.0 mm). (Blue line indicates the transplanted DMEK graft. Black line shows the measurement point of PCT [around 9.0 mm].) At the early phase after DMEK [within 1 month], peripheral edema was occasionally detected in the peripheral cornea as bullae (c) or as an irregular epithelium (d). (Arrows show corneal epithelial edema in the peripheral cornea, where DMEK graft does not cover. Circle indicates an image of transplanted graft. Broken circle and arrow indicate the measurement point of PCT [around 9.0 mm]).’ (Line 295-302)

- ‘Although it is uncertain whether the corneal edema could improve beyond the edge of the graft, there is a hypothesis that transplanted endothelial cells could migrate to the peripheral area without the Descemet membrane.^27^ Since all measurement points of the PCT (9.0 mm) were always along the outer rather than the edge of the graft, we could not evaluate the direct effect after DMEK. This problem could be one limitation of this study.’ (Line 288-292)
