# Supplementary material for: Optical characteristics after Descemet membrane endothelial keratoplasty: 1-year results
Source: PLoS One. 2020 Oct 14;15(10):e0240458. doi: 10.1371/journal.pone.0240458 (PMC7556451; doi:10.1371/journal.pone.0240458)
Supplement: Attachment — Submitted filename: Response_to_Plos_one_DMEK_HOA_2nd_revision.docx [file pone.0240458.s008.docx]

**Reviewer #2: Thank you for all your thoughtful responses and changes. Please see my suggestions below.**

- Thank you very much for your review of our work. Your comments have helped us to significantly improve our manuscript.

**Abstract**

**-Line 57: delete comma after "mean age"**

- Following your suggestion, we have deleted the comma. (Line )

**Introduction**

**- Would recommend shortening introductory paragraphs. May consider something as below:**

**"****Corneal transplantation has evolved significantly since the first full-thickness keratoplasty was performed by Dr. Eduard Zirm in 1905.** **For patients with endothelial dysfunction, a partial-thickness endothelial keratoplasty has now become standard of care.** **In particular, Descemet's stripping endothelial keratoplasty (DMEK), developed by Dr. Gerrit Melles, has resulted in the best visual outcomes for these patients.** **In this procedure, the diseased endothelium and Descemet's membrane are replaced in an anatomically precise fashion."**

- Following your suggestion, we have revised the introduction as follow.

‘	Corneal transplantation has evolved significantly since the first full-thickness keratoplasty was performed by Dr. Eduard Zirm in 1905 [1]. However, because of injury, this technique is associated with disadvantages such as graft rejection, glaucoma (steroid-dependent) and suture-related problems, slow and low visual recovery with heavy astigmatism, infection, or global rupture [2]. For patients with endothelial dysfunction, a partial-thickness endothelial keratoplasty has now become standard of care [3-5].

In particular, Descemet's membrane endothelial keratoplasty (DMEK), developed by Dr. Gerrit Melles, has resulted in the best visual outcomes for these patients [6]. In this procedure, the diseased endothelium and Descemet's membrane are replaced in an anatomically precise fashion.’ (Line 70-79)

**Results**

**- Line 229: need space between "(p<0.001)postoperatively"**

- Following your suggestion, we have revised the description as follow.

‘(p<0.001) postoperatively’ (Line 225-226)

**- Line 247: same as above**

- Following your suggestion, we have revised the description as follow.

‘(p < 0.001) postoperatively’ (Line 244)

**- Line 250: same as above**

Following your suggestion, we have revised the description as follow.

‘(p < 0.001) postoperatively’ (Line 247)

**Discussion**

**- Line 269: need space between "controls;this"**

Following your suggestion, we have revised the description as follow.

‘controls; this’ (Line 266)

**- Lines 271 and beyond: I think this section still needs work to provide a clear explanation and narrative. The following paragraphs are suggestions only - please do not feel obligated to use. However I hope it demonstrates a clearer "flow" to this section.**

**"****During DMEK, the central endothelium is stripped and replaced with an under-sized graft containing healthy endothelial cells. Post-operatively, these transplanted cells may migrate into the periphery to fill the area between the edge of the graft and the area of stripping, and come to a halt at the native peripheral endothelium due to contact inhibition.**

**This resultant discrepancy between high cell density in the center and lower cell density in the periphery may account for the persistent changes between the post-DMEK eye and a healthy control. As the central cornea deturgesces, the CCT normalizes but the PCT does not. Since the deturgescence occurs mostly via the posterior stroma, the posterior KV improves while the anterior KV remains unchanged.**

**This center-to-periphery discrepancy in cell density may be especially highlighted in this patient population of largely pseudophakic bullous keratopathy. Whereas Fuchs endothelial dystrophy preferentially affects central endothelium, PBK is thought to result in a more global loss of endothelial cells.**

**The strength of this study lies in its prospective design and meticulous post-operative follow-up without any missing data. The limitations include an ethnically limited patient population (all Japanese) with high predominance of pseudophakic bullous keratopathy with epithelial changes, which may indicate more severe disease than a patient with Fuchs dystrophy. In addition, although many of our findings are statistically significant, the clinical significance of such small differences are unknown. Lastly, backscatter and corneal haze were not evaluated because AS-OCT is not equipped to measure backscatter or densitometry.**

**This study provides valuable information regarding the long-term post-operative outcomes after DMEK. While the clinical results have been overall excellent, and certainly superiorly to full-thickness PKP and DSAEK, even after 1 year post-operatively these eyes are not comparable to healthy controls. We hypothesize these persistent changes may be a result of an irregular center-to-peripheral distribution of endothelial cells."**

- Following your suggestion, we have revised the discussion.
- ‘A thorough consideration of the corneal curvature is important to explain these findings. During DMEK, the central endothelium is stripped and replaced with an under-sized graft containing healthy endothelial cells. Post-operatively, these transplanted cells may migrate into the periphery to fill the area between the edge of the graft and the area of stripping, and come to a halt at the native peripheral endothelium due to contact inhibition. This resultant discrepancy between high cell density in the center and lower cell density in the periphery may account for the persistent changes between the post-DMEK eye and a healthy control. As the central cornea deturgesces, the CCT normalizes but the PCT does not. Since the deturgescence occurs mostly via the posterior stroma, the posterior KV improves while the anterior KV remains unchanged.’ (Line 268-276)
- ‘Since DMEK eyes may occasionally show central thinning [15, 27, 28], we also evaluated the corneal thickness in peripheral cornea at a 9.0-mm diameter (PCT). We found that both CCT and PCT gradually improved postoperatively, and CCT returned to normal thickness at 12 months, which indicated that corneal thickness remodeling occurred over time after DMEK. This might be attributable to migration of corneal endothelial cells to the peripheral area. The center-to-periphery discrepancy in cell density may be especially highlighted in this patient population of largely pseudophakic bullous keratopathy. Whereas Fuchs endothelial dystrophy preferentially affects central endothelium, PBK is thought to result in a more global loss of endothelial cells. In fact, in our study, 21 of 30 DMEK cases (70%) were PBK. The high percentage of PBK in our cohort may contribute to the findings that PCT did not normalize after surgery. It is noteworthy that corneal HOAs were reduced at 6 to 12 months after DMEK, which coincided with improvement of PCT at 12 months. Thus, we consider that the assessment of PCT would be a clinically relevant parameter when evaluating improvement in corneal edema after DMEK.’ (Line 313-326)
- ‘The strength of this study lies in its prospective design and meticulous post-operative follow-up without any missing data. The limitations include an ethnically limited patient population (all Japanese) with high predominance of pseudophakic bullous keratopathy with epithelial changes, which may indicate more severe disease than a patient with Fuchs dystrophy. In addition, although many of our findings are statistically significant, the clinical significance of such small differences are unknown. Lastly, backscatter and corneal haze were not evaluated because AS-OCT is not equipped to measure backscatter or densitometry.
- This study provides valuable information regarding the long-term post-operative outcomes after DMEK. While the clinical results have been overall excellent, and certainly superiorly to full-thickness PKP and DSAEK, even after 1 year post-operatively these eyes are not comparable to healthy controls. We hypothesize these persistent changes may be a result of an irregular center-to-peripheral distribution of endothelial cells.’ (Line 337-348)
