# Supplementary material for: Optical characteristics after Descemet membrane endothelial keratoplasty: 1-year results
Source: PLoS One. 2020 Oct 14;15(10):e0240458. doi: 10.1371/journal.pone.0240458 (PMC7556451; doi:10.1371/journal.pone.0240458)
Supplement: Attachment — Submitted filename: Response_to_Plos_one_DMEK_HOA_2nd_revision.docx [file pone.0240458.s009.docx]

*Reviewer #2:*

- Thank you very much for your review of our work. Your comments have helped us to significantly improve our manuscript.

*Introduction*

*- Line 71: not sure what you mean by "because of injury". I would delete this. The sentence works without it: "However, this technique is associated with..."*

- Following your suggestion, we have deleted the word. (Line 71)

‘However, this technique is associated with disadvantages such as graft rejection, glaucoma (steroid-dependent) and suture-related problems, slow and low visual recovery with significant astigmatism, infection, or risk of globe rupture with trauma [2].’ (Line 71-74)

*- Line 73: replace "heavy" with "significant"*

- Following your suggestion, we have revised the description. (Line 73)

‘However, this technique is associated with disadvantages such as graft rejection, glaucoma (steroid-dependent) and suture-related problems, slow and low visual recovery with significant astigmatism, infection, or risk of globe rupture with trauma [2].’ (Line 71-74)

*- Line 74: replace "global rupture" with "**risk of globe rupture with trauma"*

- Following your suggestion, we have revised the description. (Line 74)

‘However, this technique is associated with disadvantages such as graft rejection, glaucoma (steroid-dependent) and suture-related problems, slow and low visual recovery with significant astigmatism, infection, or risk of globe rupture with trauma [2].’ (Line 71-74)

*- Line 81: need to define DSAEK acronym*

- Following your suggestion, we have added the abbreviation. (Line 81)

‘Previous studies have proved the superiority of DMEK to either Descemet’s stripping automated endothelial keratoplasty (DSAEK) or ultra-thin DSAEK in terms of visual function [12-16].’ (Line 81-83)

*- Line 85: need space between "withcorneal"*

- Following your suggestion, we have revised the mistake. We are very grateful to you for the suggestion. (Line 86)

‘Previous reports have described factors associated with visual function and the specific changes in the posterior cornea after DMEK [12-16], with corneal backscatter and higher-order aberrations (HOAs) as main factors [12-20].’ (Line 85-87)

*Materials and Methods*

*- Line 106: would change "ocular surface disease" to "ocular disease". I presume patients with glaucoma or retinal pathology were excluded due to effect on VA*

- Following your suggestion, we have revised the description. (Line 107)

‘Age-matched phakic eyes without history of ocular surgery or ocular disease were selected as healthy controls.’ *(Line 106-107)*

*Results*

*- Line 160: need to define FECD acronym*

- Following your suggestion, we have revised the description. (Line 161)

‘Fuchs endothelial corneal dystrophy (FECD, n = 9)’ (Line 160-161)

*Table 1*

*- Would use PBK acronym/definition instead of BK to keep consistent with text*

- Following your suggestion, we have revised the description to keep consistent with all the manuscript. (Table 1)

*Table 2/3*

*- Would make text consistent between "Post-operative month 1" and "1 month after surgery"*

*- Could also consider shortening to Post-op month 1 or POM #1 for ease of reading the table, and then defining the acronym in the table legend/description*

- Following your suggestion, we have revised the description to the style of ‘Post-op month ~‘ with the acronym in the table legend. (Table 1)

*Discussion*

*- Lines 268-327: these paragraphs have redundant/repeating elements - they all discuss discrepancy between central and peripheral endothelial cell density and how this may explain your results. The content of these paragraphs should be re-written so that each concept is discussed once and thoroughly.*

*"**During DMEK, the central endothelium is stripped and replaced with an under-sized graft containing healthy endothelial cells. Post-operatively, these transplanted cells may migrate into the periphery to fill the area between the edge of the graft and the area of stripping, and come to a halt at the native peripheral endothelium due to contact inhibition. <>. This resultant discrepancy between high cell density in the center and low cell density in the periphery may account for the persistent changes between the post-DMEK eyes and healthy controls. As the central cornea deturgesces, the CCT normalizes but the PCT does not. <>. Since the deturgescence occurs mostly via the posterior stroma, the posterior KV improves while the anterior KV remains unchanged. <>. <> <>".*

- Following your suggestion, we have shortened the discussion. (Line 269-273)

*Figure 2:*

*- Would modify (b) to more accurately reflect size of descemetorhexis and what you discuss in your text. A 9 mm descemetorhexis on an 11 mm cornea still leaves a peripheral population of native endothelial cells.*

*- Would maybe add an additional diagram showing how you hypothesize these centrally transplanted enothelial cells migrate to the periphery and result in a center-to-periphery discrepancy*

- Following your suggestion, we have already revised the Figure 2, and discussed our hypothesis in the discussion.

*Figure 3:*

*- To emphasize and quantify the change in parallelism, I would add measurement bars of the CCT and PCT and show how the ratio defers between normal and post-DMEK eyes*

- Following your suggestion, we have added the abbreviations and bars of CCT and PCT in Figure 3.
